# Commentary: Sport activities for children and adolescents: the position of the European Academy of Paediatrics and the European Confederation of Primary Care Paediatricians 2023—part 1: pre-participation physical evaluation in young athletes

**DOI:** 10.3389/fped.2026.1917687

**Published:** 2026-08-19

**Authors:** Abdullah Akkuş

**Affiliations:** Department of Pediatrics, Faculty of Medicine, Necmettin Erbakan University, Konya, Türkiye

**Keywords:** child, paediatric athlete, Paediatric Sports Medicine, physical activity, pre-participation evaluation

## 1 Introduction

We read with great interest the position statement published by Turska-Kmieć and colleagues on behalf of the European Academy of Paediatrics (EAP) and the European Confederation of Primary Care Paediatricians (ECPCP) (1). As a group attempting to establish the Division of Paediatric Sports Medicine in Türkiye, we commend the authors for producing a comprehensive and clinically valuable framework for pre-participation physical evaluation in young athletes. However, we wish to draw attention to a terminological inaccuracy in the classification of sports types presented in the Definitions section of the manuscript, which, in our view, merits correction.

### 1.1 A terminological inaccuracy in the classification of physical activity types

The authors present the following typology: “There are various types of sport activity which can be defined under different settings such as: Leisure sports, Competitive sports, Exercise training.” They attribute this classification to the WHO glossary of terms. This attribution is factually incorrect and conflates two fundamentally distinct conceptual frameworks—the WHO domain classification of physical activity and the typological classification of sports by organisational structure.

According to the WHO's established framework, physical activity is classified by the domain in which it occurs, not by the type of sport. Physical activity can be performed as part of work, domestic chores, transportation, or during leisure time (2). The four canonical domains of physical activity are occupational, domestic/household, transportation, and leisure time (2, 3). Within this framework, the leisure domain includes voluntary activities undertaken during free time for enjoyment, relaxation, or health benefits, such as jogging or swimming (3). Critically, the WHO defines “leisure-time physical activity” as a discretionary activity performed when one is not working, transporting oneself, or doing household chores, with walking and recreational games cited as examples of this domain (4). The term “leisure sports,” as used in the manuscript, does not appear in the WHO glossary of terms and does not represent a WHO-recognised category. What the WHO framework describes is “leisure-time physical activity”—a domain descriptor denoting the context in which activity occurs, not a type of sport. “Leisure-domain physical activity” refers to activities including sports participation, exercise conditioning or training, and recreational activities (2–4). In other words, sport and non-competitive recreational activity can both occur within the leisure domain; these are not mutually exclusive categories, and “leisure sports” is not a meaningful subcategory recognised by the WHO.

The WHO framework for physical activity classification is not merely one framework among many: it is the globally authoritative and institutionally endorsed standard against which population-level physical activity terminology is benchmarked. When a position statement published under the aegis of the European Academy of Paediatrics attributes an alternative classification to the WHO without adequate sourcing, it risks introducing terminological ambiguity into a clinical guidance document intended for practitioners across multiple European countries. In this context, citation accuracy and conceptual precision are inseparable.

It might be argued that similar distinctions between physical activity types have been described in previous paediatric sports cardiology literature, such as in Takken et al. (5). However, the full title and content of this reference—“Recommendations for physical activity, recreation sport, and exercise training in paediatric patients with congenital heart disease”—does not employ the term “leisure sports.” The distinction between “recreation sport” and “leisure sports” is not merely semantic: Recreational sport refers to the organisational or competitive character of a sporting activity, whereas leisure sports, as used in the original position statement, implies a WHO domain classification that, as demonstrated earlier, does not exist. The Takken et al. (5) framework was developed specifically for a clinical subpopulation—children with congenital heart disease—to guide risk stratification in that context, and does not purport to provide a general WHO-endorsed taxonomy of physical activity types applicable to the broader paediatric population addressed by the EAP/ECPCP position statement. More broadly, the existence of disease-specific or clinical subpopulation frameworks that distinguish between sport participation types does not validate the attribution of such frameworks to the WHO when no such attribution is warranted.

### 1.2 Clinical and epidemiological implications

The distinction is clinically and epidemiologically significant. A child competing in a registered football academy and a child playing football recreationally on weekends may both be engaging in “leisure-domain physical activity” under the WHO classification. However, their pre-participation evaluation needs, injury risk profiles, and the applicable clinical guidelines differ substantially. Conflating the WHO domain framework with a sport typology may inadvertently suggest that non-competitive recreational activity falls outside the scope of clinical oversight—a conclusion that the position statement itself does not intend and one that would be contrary to the recommendations of the EAP and ECPCP.

## 2 Discussion

We respectfully suggest that the authors consider revising the relevant section in a potential corrigendum or future update. A more accurate formulation—aligned with both the WHO framework and the clinical objectives of the position statement— might read as follows: “Physical activity in children may occur across multiple domains (occupational, transportation, domestic, and leisure) and in multiple organizational contexts, including structured exercise training, unstructured recreational activity, and sports. For the purposes of clinical sports PPE, all children participating in regular, structured physical training—regardless of competitive level—should be considered for evaluation.”

We also propose that the terminological ambiguity identified in the original position statement reflects a broader structural issue: The classification of physical activity types and the organisation of sport participation for children fall primarily within the domain of Paediatric Sports Medicine—a subspecialty whose core competency encompasses developmental physiology, physical activity epidemiology, and sport-specific risk stratification across the full paediatric age range (6, 7). It has been noted that even among paediatric cardiologists, the adoption of evolving sport classification models may result in confusion and unanticipated delays in clinical decision-making, underscoring the need for specialist input from those whose primary expertise lies at the intersection of physical activity science and paediatric medicine (7, 8). The terminological imprecision observed in the original position statement may, in part, reflect the inherent challenge of addressing physical activity classification from outside this subspecialty's core knowledge base—an observation we raise not as a criticism of the authors, whose contribution to the field is substantial, but as an argument in favour of greater involvement of Paediatric Sports Medicine specialists in the development of future paediatric physical activity guidance documents in Europe.

We raise this point in the spirit of scientific precision and in recognition of the importance that the EAP and ECPCP position statements carry for paediatric clinical practice across Europe. The contribution of this document to the field of Paediatric Sports Medicine is substantial, promoting the participation of paediatricians in the evaluation of paediatric athletes. As we advocate that paediatricians must be interested in Paediatric Sports Medicine to a greater extent, our comment is intended only to strengthen its terminological foundation.
